# Crohn’s Disease: Basic Characteristics of the Disease, Diagnostic Methods, the Role of Biomarkers, and Analysis of Metalloproteinases: A Review

**DOI:** 10.3390/life13102062

**Published:** 2023-10-15

**Authors:** Grzegorz Pasternak, Grzegorz Chrzanowski, David Aebisher, Angelika Myśliwiec, Klaudia Dynarowicz, Dorota Bartusik-Aebisher, Barbara Sosna, Grzegorz Cieślar, Aleksandra Kawczyk-Krupka, Rafał Filip

**Affiliations:** 1 Department of General Surgery, Provincial Clinical Hospital No. 2 in Rzeszów, 35-301 Rzeszów, Poland; gpasternak@ur.edu.pl; 2 Department of Biology, College of Natural Sciences, University of Rzeszów, 35-310 Rzeszów, Poland; 3 Department of Photomedicine and Physical Chemistry, Medical College, University of Rzeszów, 35-310 Rzeszów, Poland; 4 Center for Innovative Research in Medical and Natural Sciences, Medical College, University of Rzeszów, 35-310 Rzeszów, Poland; amysliwiec@ur.edu.pl (A.M.); kdynarowicz@ur.edu.pl (K.D.); 5 Department of Biochemistry and General Chemistry, Medical College, University of Rzeszów, 35-310 Rzeszów, Poland; dbartusikaebisher@ur.edu.pl; 6 Department of Internal Medicine, Angiology and Physical Medicine, Center for Laser Diagnostics and Therapy, Medical University of Silesia in Katowice, Batorego 15 Street, 41-902 Bytom, Poland; barbara.sosna@hotmail.com (B.S.); gcieslar@sum.edu.pl (G.C.); akawczyk@gmail.com (A.K.-K.); 7 Department of Internal Medicine, Medical College, University of Rzeszów, 35-310 Rzeszów, Poland; rfilip@ur.edu.pl

**Keywords:** Crohn’s disease, characteristics, diagnostics, metalloproteinases

## Abstract

Crohn’s disease is a chronic inflammatory bowel disease that affects the ileum and/or large intestine. At the same time, it can also affect any other part of the human body, i.e., from the mouth to the anus. In Crohn’s disease, the physiology and functioning of the epithelial barrier are inhibited due to the correlation of various factors, such as the environment, genetic susceptibility or intestinal microbiota. The symptoms are very troublesome and cause a significant reduction in quality of life, sometimes occurring with paralyzing permanent damage to the digestive tract, requiring enteral or parenteral nutrition throughout life. In order to make a proper and accurate diagnosis, an appropriately selected diagnostic path in a given clinical entity is necessary. Standard diagnostic methods are: laboratory examination, histopathological examination, endoscopic examination, X-ray, computed tomography, ultrasound examination and magnetic resonance imaging. Medical biology and the analysis of metalloproteinases have also proved helpful in diagnosing changes occurring as a result of Crohn’s disease. Here we provide a thorough review of the latest reports on Crohn’s disease and its genetic conditions, symptoms, morphology, diagnosis (including the analysis of Crohn’s disease biomarkers, i.e., metalloproteinases) and treatment.

## 1. Introduction

Crohn’s disease (CD) is a chronic inflammatory bowel disease with possible recurrences [1,2]. It is a subtype of inflammatory bowel disease (IBD), a chronic inflammatory disorder of the gastrointestinal tract. Treatment of this condition is lifelong [3,4]. The reason for its post-onset is a transbronchial inflammation, which can affect any part of the gastrointestinal tract. Its phenotype is usually variable and is determined either by the risk of progression or by the location of the disease [5].

Crohn’s disease cannot be fully cured, but, with appropriate diagnostic and therapeutic procedures, emerging inflammatory changes can be controlled [6]. This disease can occur at any age; however, it is most often diagnosed between the ages of 15 and 30, i.e., in young people in the second and third decade of life [7]. People over the age of 65 may also develop some symptoms. It is estimated that, in the European Union, 40 to 50 people per 100,000 inhabitants suffer from Crohn’s disease.

Inflammatory bowel diseases (IBD) are a group of diseases of unexplained etiology and chronic course, with periods of exacerbation and remission [8]. Initially described in the 19th and early 20th centuries, individual cases from Great Britain and Northern Europe grew in number, and geographically expanded, so that today they are recognized all over the world, and their numbers are not decreasing. A characteristic feature of this group of diseases is the continuity of inflammatory changes in the mucosa starting in the rectum and covering the ascending large intestine with possible involvement of the final segment of the small intestine. The relationship between the disease and the immune system remains undeniable. Clinically, IBD carries many intestinal complications and systemic diseases, as well as dysplastic changes—the risk of developing colorectal cancer [9].

Inflammatory bowel disease is most common in Scandinavia, Western Europe and North America. Currently, studies indicate that the incidence of IBD is lower in developing countries. The incidence of IBD has been increasing worldwide for about 20 years. Some Western countries, such as Canada, predict that there will be an increase of nearly 33.4% between 2015 and 2025 [10].

According to data compiled by Ng et al., developing countries such as Romania and Turkey have an incidence rate of ulcerative colitis of approximately 2.42–21.00 per 100,000 people. However, in developed countries (such as Italy, Portugal, Switzerland, Spain and Great Britain) this coefficient ranges from 44.4 to 198.00 per 100,000 people. In some European countries (Germany, the Netherlands, Denmark, Norway, Sweden and Finland) this ratio is even higher than 198.00 per 100,000 people [11]. In Asian populations, the incidence ranges from 5.3 to 63.6 per 100,000 people, while in North America it ranges from 37.5 to 238 per 100,000 people [12]. It has been noted that in Europe there is a geographical gradient in the prevalence of IBD with higher rates in the north and lower rates in the south.

Most IBD patients in Europe and North America are in the 30–40-year age group at diagnosis. It has been observed that the average age at diagnosis tends to be slightly higher in Asian countries compared to Western countries, and a study by Souza in Southeast Brazil showed that there is a trend towards a second peak in new hospital admissions in the 60–69-year age group because of IBD [13].

The etiology of IBD remains unexplained. There are genetic hypotheses, hypotheses of increased permeability of the mucosa for antigens from the intestinal lumen and hypotheses regarding the ongoing inflammation of small vessels of the mucosa leading to altered mucosal permeability. Certainly, the immune system plays the most important role in the pathogenesis of the disease [14]. The aim of this review is to characterize the latest reports on Crohn’s disease and its genetic conditions, symptoms, morphology, diagnosis (including the analysis of Crohn’s disease biomarkers, i.e., metalloproteinases) and treatment.

## 2. Characteristics of Crohn’s Disease

### 2.1. Genetic Factors

Differences in the incidence of Crohn’s disease (CD) by race and ethnicity indicated, from the outset, that genetic factors play an important role. An increased incidence of CD is observed among family members of patients, especially among first-degree relatives. In this group, the incidence is 10–30% higher than in the general population, with no increased incidence among spouses, which argues against the involvement of infectious agents [15]. A higher incidence of the disease has been described in identical twins. A higher prevalence of DR2HLA II, DR9 and DRB10 alleles has been described in patients with CD. Predisposition to the development of the disease is also associated with the presence of certain regions in chromosomes 2 and 6 and regions located in chromosomes 3, 7 and 12 [16]. Another element of risk is mutations that contribute to loss of function in the NOD2 allele. They are responsible for stenosis. Currently, there is still a lack of sufficient research characterizing the basis of pathogenicity of NOD2 mutations [17]. The three most common Crohn’s disease-related mutations in NOD2 are Arg702Trp, Gly908Arg, and Leu1007fsinsC, which play an essential role in recognizing and sensing microbes. By binding microbial ligands, NOD2 activates the transcription factor nuclear factor-κB and positively regulates the host’s innate immune defense [18]. According to Siddique et al., who analyzed epidemiological studies that suggested that genetic factors play a significant role in determining susceptibility to CD, the most commonly associated gene is NOD2/CARD15. Research shows that polymorphisms in the NOD2 gene contribute to the inhibition of the function of the NOD2/CARD15 protein, impairing the inflammatory response to external stimuli. These polymorphisms alter protein structure at the domain or adjacent region level, disrupting bacterial recognition and increasing the production of IL-12, IL13, IL23 and other pro-inflammatory cytokines, leading to chronic inflammation [19].

### 2.2. Environmental Factors

Nutritional deficiencies occur in 20–85% of CD patients, with protein–energy malnutrition being the most common. There are also overweight IBD patients; they have a higher colectomy rate than eutrophic patients, increased need for permanent ileostomy, longer hospital stays, higher rates of incisional hernia after ileo-rectal anastomosis and increased risk of non-alcoholic fatty liver disease and thrombotic disease [20]. Increased body weight is also associated with early loss of response to drugs that treat CD. The formation of pathogenic microflora in genetically predisposed individuals is associated with changes in epithelial function, dysregulation of the immune function of the digestive tract and persistent inflammation of the intestines [21].

### 2.3. Symptoms

The symptoms of CD range from rectal and anal irritation to frequent and profuse bloody diarrhea accompanied by colic pains in the middle and lower abdomen, especially on the left side [22]. Profuse gastrointestinal bleeding may occur in 3% of patients. There are also cases of constipation.

In 1.6–6% of patients, megacolon toxicum develops; in this form atony, intestinal dilatation and thinning of its wall are accompanied by a septic state resulting from the penetration of bacteria from the intestinal lumen into the blood. Complications of severe CD include perforations, peritonitis and massive gastrointestinal bleeding [23]. Other types of complications are shortening and narrowing of the intestinal lumen and developing dysplasia, which increases the risk of developing colorectal cancer. Colorectal cancer is most common in patients after the seventh year of the disease. The greatest risk concerns patients with pancolitis—25% after 20 years. In comparison, 3.7% of patients with rectal and sigmoid colon involvement develop colorectal cancer after 20 years of disease [24].

Patients with CD have a wide spectrum of extraintestinal symptoms. These include skin and mucosal symptoms—erythema nodosum and erythema multiforme, gangrenous dermatitis, skin infections, inflammation of the corners of the mouth and aphthae of the oral mucosa; eye symptoms—conjunctivitis, choroid and iris inflammation; joint symptoms—arthritis, ankylosing spondylitis, sacroiliitis; cardiovascular symptoms—pericarditis, vasculitis, aneurysms; anemia; vascular thrombosis; obstructive lung disease; sclerosing cholangitis; hepatitis; cirrhosis; pancreatic stenosis and cholangiocarcinoma; gallstones; urolithiasis; perirenal abscesses; and amyloidosis. Extraintestinal symptoms have been shown to be associated with more advanced disease and poorer prognosis [25].

Crohn’s disease may be recurrent and persist for many years. The therapeutic measures taken only prevent further development by inhibiting metabolic activity [5]. Therefore, there is a high probability that this disease will cause complications in various organs. The severity, type and number of complications vary in many respects and depend on various factors. The most common causes of complications are eating disorders, which occur in almost all cases, and those related to long-term treatment. Additionally, pharmacological or surgical treatment may also cause side effects. Complications may involve such systems of the human body as:

- digestive system;
- osteoarticular system;
- circulatory system;
- the nervous system;
- excretory system;
- reproductive system.

The most common complication of Crohn’s disease is ileitis [26]. It manifests itself with severe abdominal pain, bleeding and diarrhea. Untreated inflammation has widespread consequences, including permanent impairment of intestinal physiology [26]. Other types of complications of Crohn’s disease include: anterior uveitis, cholelithiasis, scleritis and episcleritis, nephrolithiasis, osteoporosis and venous thromboembolism [27]. Each of the emerging complications poses a threat to the patient’s health and life. For example, venous thromboembolism may cause pulmonary embolism, leading to partial or complete occlusion of the pulmonary arteries.

Each emerging disease brings with it so-called “side effects” in the form of more or less serious complications. The list of complications of Crohn’s disease is constantly expanding. To minimize the risk of their formation, the appropriate form of treatment is crucial, which depends on an accurately performed diagnostic analysis and an appropriately selected diagnostic tool.

### 2.4. Morphology

CD is characterized by diffuse and continuous inflammation limited to the colonic mucosa. Macroscopically, the inflamed mucosa is swollen, granular, friable, reddened and bleeds easily, with or without ulcerations of various sizes [28]. Mucosal changes include continuity from the rectum, proximal along a varying length of colon, with the distal colon likely to be more inflamed than the proximal. The regenerating mucosa shows signs of atrophy, the adjacent unchanged areas are thicker and protrude above the areas with atrophy, which gives the appearance of pseudopolyps. The histological picture is different in the active phase of inflammation and in remission [29]. In the full-blown phase, the inflammation covers the mucous membrane and blood vessels. There is a profuse inflammatory infiltrate made of lymphocytes, plasma cells, and macrophages with associated infiltration from neutrophils, less numerous eosinophils and mast cells [30]. Granulocytes occur both in the lamina propria and penetrate into the crypts, creating micro-abscesses. Ulcers covered with granulocytic infiltration form above the inflamed crypts. In CD, the mucosa may return to normal or remain atrophic during remission [31]. Subsequent recurrences lead to disorders of the architecture of the glands in the form of shortening of the crypts and the presence of spaces between the lower part of the crypts and the upper edge of the muscular layer of the mucosa, as well as branching of the crypts, persistent chronic inflammatory infiltration with hyperplasia of the intestinal lymphatic system and an increased number of plasma cells at the base of the membrane mucosa. The presence of Paneth cells in the mucosa of the left colon is a metaplastic process also associated with chronic damage to the crypt epithelium [32]. Metaplasia of the pyloric glands is rare. In fulminant cases, necrosis and ulceration occur, which involve the submucosa and muscularis. In cases of long-term relapses and remissions, the intestinal wall may also be significantly shortened, with a dominant picture of fibrosis of the inner layers of the wall [33].

One of the disabling manifestations of CD is perianal fistulas, which in many cases are associated with rectal disease [34]. The hallmarks of ulcerative colitis are bursting ulcers, granulomatous inflammation and submucosal fibrosis. Histologic features of CD include distortion of the crypts, lymphocyte infiltration and chronic inflammation of the rectum, mostly limited to the lamina propria [35,36,37]. Current therapeutic options for CD perianal fistulas are very limited and include antibiotics, immunomodulators, biologics and mesenchymal stem cells [38]. However, these therapies are combined with chirurgical methods. Thus, surgical removal is the primary method of treating fistulas, although it is not a complete cure for the disease [39]. The etiopathogenesis of perianal fistula in CD is currently unknown. The diseased mucosa is destroyed and ulcerated and then replaced by degranulation tissue. It can be assumed that inflammatory cells and myofibroblasts are interfered with, trying to repair the damaged tissue by depositing collagen or other components of the extracellular matrix (ECM). All this leads to a remodeling of the tissue, which is characterized by high stiffness [40,41]. Fistulas in Crohn’s disease are likely formed by epithelial–mesenchymal transition (EMT) of intestinal fibroblasts, which impairs the ability of the above cells to repair any damage to the mucosa [42]. Under-going EMT, intestinal epithelial cells acquire properties of an invasive and migratory nature and, above that, lose both cell polarity and cell-to-cell contact. This leads to them becoming myofibroblasts [43,44]. However, the molecular mechanisms triggering EMT still remain to be investigated. Both CD and ulcerative colitis (UC) are two major types of non-specific inflammatory bowel disease that share common pathological and clinical features. However, they have several significant differences. The clinical diagnosis of CD is usually established based on a collective evaluation of the clinical picture and findings from endoscopy, histopathology and radiography [45,46]. There is a need for objective clinical differentiation of CD and UC in patients who have inflammatory bowel disease in order to develop a treatment plan [47,48,49,50]. However, the differential diagnosis of the above subtypes still remains a major clinical challenge due to the fact that there is no single diagnostic modality for UC or Crohn’s colitis [49,50,51]. Based on the literature, about 5%–15% of patients do not meet strict guidelines for the diagnosis of UC or CD [52], and for about 14% of patients diagnoses change over time [53,54].

#### Molecular and Cellular Aspects of Crohn’s Disease

In CD, there is infiltration of inflammatory cells or damage to epithelial cells in the intestinal mucosa. The reconstruction process involves cells, factors or molecules that play a key role in the repair of damaged epithelium [55]. Crohn’s disease patients are characterized by increased TLR4 expression. Increased expression of the CD14 receptor is also noted in selected parts of the intestine. Deregulated expression of TLR receptors, especially TLR4 and CD14, in various parts of the intestinal mucosa is crucial in the morphology of CD patients. This is related to the process of interaction of the CD14 membrane protein with LPS, which leads to the activation of TLR4 and, with the involvement of two indirect signaling pathways, causes the expression of NF-κB, thereby inducing the expression of TNF-α and IL-6. Another molecular aspect of CD is the imbalance between pro-inflammatory and anti-inflammatory cytokines associated with antigen-presenting cells (APCs), Th1, Th2, Treg, Th17 lymphocytes, dendritic cells (DC) and macrophages. Examples of these cytokines are: TNF-α, IL-1, IL-4, IL-5, IL-6, IL-10, IL-12, IL-13, IL-17, IL-18, IL-21, IL-23, IL-27 and IFN-γ and TGF-β. Collectively, these proteins are involved in many immune responses to IBD, such as regulating the production of inflammation. In CD, disorders lead to deregulation or overproduction of effector T cells involved in the development and exacerbation of the disease. There is a predominance of CD4+ T cells from the Th1 group, a phenotype responsible for, among other things, the intense the secretion of TNF-α, IL-6 and IFN-γ, which inhibit Th2 cell function and proliferation [56].

### 2.5. Criteria Used to Diagnose Crohn’s Disease and Differentiate It from Other Similar Diseases

The basic tool in the assessment of changes caused by Crohn’s disease is histopathological evaluation. All properly prepared preparations are assessed by a doctor specializing in pathology. In the verification of specific preparations, strictly defined changes are analyzed in order to standardize the identification protocol [57]. The analysis is usually performed based on the Colonic and Ileal Global Histologic Disease Activity Score (CGHAS) [58]. This indicator includes the degree of epithelial damage, changes in the structure of the intestinal walls, the number of mono- and multinucleated cells in the epithelium and the presence of inflammatory changes in the form of ulcers and granulomas [58]. The procedure analyzes changes that are the direct cause of Crohn’s disease. These are eight variables, each of which is assessed individually. Depending on the extent or occurrence of a given change, points are awarded on an appropriately prepared scale. In the final summary, the points from individual changes are added up and the final result is given. The higher the result, the stronger/more dangerous the inflammation caused by Crohn’s disease in the body. According to the point awarding procedure, you can “obtain” a maximum of 16 points.

In Table 1, a method of verifying intestinal changes in Crohn’s disease, according to the CGHAS disease activity index is presented.

### 2.6. Diagnostic Methods

In order to make a proper and accurate diagnosis, an appropriately selected diagnostic path in a given clinical entity is necessary. The tool package is always selected individually for each patient and depends on the preferences of the attending physician. The standard procedure assumes that, at the beginning, minimally invasive tests are used, with an uncomplicated course and with the fastest possible results. This process also applies to the diagnosis of Crohn’s disease. Standard diagnostic methods are: laboratory examination, histopathological examination, endoscopic examination, classic X-ray examination (X-ray), computed tomography, ultrasound examination and magnetic resonance imaging.

#### 2.6.1. Laboratory Examination of Blood and Stool

Laboratory testing of blood and stool does not allow for the direct diagnosis of inflammatory bowel disease, but it can be a kind of screening element in the diagnosis of inflammation in the patient’s body. This test is a technique for relative control of the course of Crohn’s disease in mild and asymptomatic phases [59]. Based on blood count, anemia can be determined, or the number of white blood cells (leukocytes) can be estimated. The biochemical blood test can determine the levels of CRP (C-reactive) protein, ESR, electrolyte or iron concentration, the presence of ASCA antibodies, average platelet volume, fibrinogen, neutrophil to lymphocyte ratio (NLR) and the International Normalized Ratio—INR [60]. Biomarkers of inflammatory bowel disease include C-reactive protein and fecal calprotectin. In ongoing inflammation, the level of CRP and the NLR most often attract attention.

According to Gao et al. [61], the NLR ratio may confirm the existence of inflammation caused by ongoing Crohn’s disease, but it is not an indicator of the activity of this disease. An increased content of neutrophils in the blood may result in the accumulation of bacteria in particular sections of the digestive system, leading to inflammation. Neutrophils accumulate in intestinal microvilli in the intestinal lumen, leading to the inhibition of the physiology of the intestinal monolayer epithelium [61]. Another biomarker of inflammatory change is the erythrocyte sedimentation rate (ESR). The ESR describes the sedimentation rate of red blood cells (erythrocytes) in the blood plasma [62]. In the emerging inflammatory state of Crohn’s disease, the erythrocyte sedimentation rate is higher than in healthy patients.

Laboratory tests also include stool examination aimed at detecting various types of pathogens and toxins and determining the concentration of calprotectin. Calprotectin is a heterodimer protein formed by the combination of two proteins: S100A8 and S100A9 [63]. They occur in myeloid cells, among others, and in neutrophil granules and on the surface of monocytes and macrophages [64]. In normal physiology, when there is no inflammation, calprotectin concentrations are low [63]. An increased concentration of this marker indicates intestinal inflammation, which leads to further complications and the development of subsequent clinical entities. Calprotectin appearing in feces causes lymphocytic infiltration of the mucosa [64]. Thanks to the labeling of calprotectin in stools, the basic, painful examination, such as a colonoscopy to assess intestinal inflammatory changes, is often omitted. It is worth noting that by examining the stools, the doctor may also detect signs of overt or hidden bleeding from a specific part of the digestive system. Clinical examination of blood and stool can also assess cytokine activity in Crohn’s disease [65]. Cytokines belong to a large group of peptides. They are produced by cells in the immune system. They influence the presence and activation of cells involved in the immune response. An example of such cells is fibrocytes formed from bone marrow cells [66]. The level of fibrocytes in CD is significantly increased compared to the result in a healthy patient [67]. The level of fibroblasts increases with increasing disease activity, which is why these cells are often referred to as pathobiological markers [67].

Laboratory testing of blood and stool is a, so-called, first contact. It may not directly indicate the development of CD, but it is used to confirm the existing inflammation in the patient’s body. It is the starting point for subsequent, more advanced diagnostic tools.

#### 2.6.2. Histopathology

It seems that in the era of molecular medicine, histopathological tests are a thing of the past [68]. However, this is too general an assumption. It is true that molecular medicine is a relatively “young” technique and offers a wide range of possibilities, but traditional histopathological tests have not been abandoned. According to Pai and Jairath [57], histopathology is important in Crohn’s disease and is widely used in the general assessment of activity. Histopathological examination clearly distinguishes Crohn’s disease from many other inflammatory bowel diseases and cancers.

Preparations for histopathological examination are obtained as a result of a biopsy procedure, i.e., after the so-called surgical resection of intestinal lesions [69]. Small fragments (sections) of diseased tissue are taken. They can be thoroughly analyzed using a microscope or examined with another diagnostic tool, e.g., using magnetic resonance imaging to conduct in vivo imaging and ex vivo/in vitro examination of the collected samples [70,71]. Changes that indicate inflammatory bowel disease under a microscope include:

- loss of functioning of intestinal villi, the so-called projections that cover the mucosa of the small intestine. Their most important function is the digestion and absorption of food;
- inflammatory infiltrate;
- the presence of granulomas in the mucous membrane, i.e., a collection of bacterial cells with a spherical shape, participating in the immune response;
- uncontrolled growth of lymphatic follicles, i.e., lymphocytes, which constitute a protective barrier against microorganisms;
- appearance of fibrosis.

Other pathological features that classify the ongoing inflammation of Crohn’s include: inflammation of the lamina propria of the large intestine, the presence of plasmacytosis, i.e., a significant amount of plasma cells, and neutrophilic inflammation of the intestinal mucosa [57]. Under the microscope, it is also possible to assess ulcers in the small intestine, analyze dense lymphoid clusters and observe the number and size of swollen intestinal lymph nodes [72]. The evaluation of the histopathological specimen provides information about the started or ongoing inflammation and the degree of disease activity. It may also prove to be a helpful tool in later stages of the clinical proceedings [73].

#### 2.6.3. Endoscopy

Endoscopy is a series of diagnostic and therapeutic procedures that enable visualization of the inside of the patient’s body. It plays a key role in the diagnosis of inflammatory bowel diseases [74]. It is an essential tool in the diagnosis of inflammatory lesions in places that are difficult to access by other methods. According to research conducted by Lescut et al. [75], over 65% of patients with Crohn’s disease were diagnosed preoperatively by endoscopic examination. Its undeniable advantage is the ability to assess inflammation, differentiate Crohn’s disease from other inflammatory clinical entities and assess the course of treatment [74]. The key role of the tool is to visualize the internal walls of the digestive tract. In medicine, the following forms of endoscopic examination are distinguished: gastroscopy (visualization of the upper gastrointestinal tract); ileocolonoscopy (visualization of the colon and the final section of the small intestine); sigmoidoscopy (visualization of the sigmoid colon); retroscopy (visualization of the anus and rectum); colonoscopy (visualization of Bauchin’s valve); andcapsule endoscopy.

Each of the methods used allows for accurate visualization of a specific section of the digestive system. The main hardware component is the endoscope, i.e., a special medical speculum with its own light source. It has a lens system that transmits the image from inside the body to a special monitor screen used by the doctor during the procedure. During an endoscopic examination in the case of Crohn’s disease, the doctor may observe changes such as: swelling, the status of the organ’s vascularization, erythema, the degree of granulation, the presence of ulcers and polyps or the condition of the intestinal mucosa [74]. During colonoscopy and ileocolonoscopy, it is possible to analyze focal lesions and visualize furrowing of the esophageal mucosa [76]. Each endoscopic technique has extraordinary diagnostic capabilities and advantages in imaging the patient’s intestinal lesions.

A standard analytical protocol improves the assessment of inflammatory lesions. Based on Khanna et al. [77], to assess the extent of inflammatory changes in inflammatory bowel disease, the Simple Endoscopic Score for Crohn’s disease (SES-CD) is used. In this procedure, four elements are analyzed, such as: the size of the ulcer, the ratio of the area affected by ulcers to the healthy surface, the part of the area covered by inflammation and the degree of narrowing [77]. Similarly to the histopathological index, each of the analyzed lesions is assigned numerical values corresponding to the degree of lesions on a scale of 0–3. In the summary, the point total is calculated. A higher numerical value of the sum indicates a more advanced intestinal inflammation.

A modern form of endoscopic examination is the use of capsule endoscopy [78]. Capsule endoscopy is a procedure that uses a small wireless camera. It is estimated that the standard dimensions of the capsule are (26 × 11) mm, and the weight is approximately 3 g [79]. The capsule is swallowed by the patient and moves through subsequent sections of the digestive system, thanks to natural peristaltic movements, taking thousands of photos. The images are saved on a special receiver worn by the patient. As a result of the examination, a film is created which, after being downloaded from the receiver, is analyzed by the attending physician [80]. The inventor of wireless capsule endoscopy was the Israeli engineer Gavriel Iddan. This discovery is further evidence confirming the close correlation between technology/physics and medicine. This technique was officially approved by the Food and Drug Administration in 2001. The indisputable advantage of this method is that it allows the visualization of those parts of the digestive tract that are difficult to visualize with traditional tools. Capsule endoscopy is used to assess bleeding from the gastrointestinal tract and to diagnose canker sores, deep ulcers and, possibly, slight narrowing of the intestinal walls [81]. It also allows monitoring the current inflammation in the patient’s digestive system. In Crohn’s disease, capsule endoscopy can also assess ulcerative lesions in the intestine and fissure lesions.

According to Wilkins, Jarvis and Patel [76], the sensitivity of detecting lesions in capsule endoscopy is approximately 83%, while the sensitivity of colonoscopy is approximately 74%. Although capsule endoscopy has many advantages and enables the visualization of lesions that are often difficult to access with other diagnostic methods, it still has certain limitations. One of the main reasons for this phenomenon is the fear that the capsule (despite its small size) may stay in a certain section of the patient’s digestive tract. Therefore, one of the main contraindications of the examination is the presence of fistulas, numerous strictures and fragmentary intestinal obstructions [82]. Due to the possibility of the capsule remaining in the patient’s digestive system, this method is used secondarily, after a preliminary analysis of the tract using other diagnostic methods [76].

#### 2.6.4. X-ray

X-ray diagnostics is also used in the diagnosis of Crohn’s disease. In many cases, this technique is one of the first imaging methods used in acute pain conditions. The examination usually involves the administration of a contrast agent. In the literature, the technique in which the contrast agent is administered through a nasoenteric tube is called conventional enteroclysis [83]. The nasoenteric tube takes the form of a probe that is placed in the intestine, traveling through the digestive tract and stomach directly to the loop of the small intestine. A contrast solution containing barium is administered through the inserted probe [84]. The contrast allows for better visualization of changes in the intestinal walls. Moreover, based on the results obtained, it is possible to assess ulcers, changes in the intestinal mucosa and visualize fistulas. The X-ray image is classified based on the assessment of four main parameters [85]. The following is analyzed:

the extent of inflammation;

presence and assessment of fistulas and perforations;

presence of fibrostenotic subtype;

presence of the repair/regenerative subtype.

The appearance of superficial or deep ulcers may indicate the severity of intestinal inflammation [85]. In standard X-ray enteroclysis, the radiologist can also assess irregularities in the area of the ileocecal valve. According to Masselli, Vecchioli and Gualdi [86], radiological assessment also takes into account the degree of change in places that cannot be assessed in cross-sectional radiographs. An example is the assessment of the area between the ligament of Treitz and the ileocecal valve. The sensitivity of classical enteroclysis is approximately 98% [83]. The examination also allows for verification of intestinal peristalsis, mainly thanks to the administration of a contrast agent, and analysis of intestinal transit. Thanks to the ability to visualize the contrast flow process, the radiologist can estimate the physiological condition of the intestinal loops [83].

#### 2.6.5. Computed Tomography

Contrast examination of the intestines using computed tomography is called CT enterography (CT). This technique was introduced as an extended option to conventional X-ray enteroclysis [83]. The test allows for quantitative assessment and identification of structural damage and verification of Crohn’s disease activity in the small intestine [87]. Its main advantage is its lack of invasiveness. The sensitivity of this test to any changes and disease activity is approximately 97.8% [88]. CT enterography has extraordinary spatial resolution, thus enabling the acquisition of a multiplanar 3D profile [89]. The examination requires the administration of a contrast agent that visualizes the lumen and the walls of the intestine from the inside [90]. The contrast agent is an iodine-based solution. Table 2 shows typical changes analyzed in a study using computed tomography.

The method of computed tomography enterography can detect and visualize inflammation in areas of the abdominal cavity that are inaccessible to standard endoscopy or conventional enteroclysis. Without computed tomography imaging of the digestive tract, it is not possible to decide whether surgery is necessary or to determine the form of surgery [91]. Computed tomography is characterized by: good image quality, widespread availability (much easier compared to, for example, magnetic resonance imaging) and quite easy to use.

Despite numerous advantages, computed tomography also has disadvantages, which makes it quite a risky option in some cases. One of the disadvantages is the patient’s exposure to high doses of ionizing radiation. The number of patients who are children and adolescents is increasing every year. Therefore, the use of tomography must be kept to a relative minimum. Instead of computed tomography, an ultrasound examination can be used, which does not use X-rays, making it completely safe for the youngest patients.

#### 2.6.6. Ultrasonography

Ultrasonography (USG) is a non-invasive test, available in almost every medical facility. It is characterized by a relatively uncomplicated test procedure and, most importantly, it is free from any dangerous side effects. According to Sarno et al. [92], intestinal ultrasound has become more common over the last few years, which has increased its importance in the diagnosis of inflammatory bowel diseases. Due to its numerous advantages, this examination is as valuable as a complex computed tomography examination. Compared to computed tomography, it does not expose the patient to ionizing radiation. When examining the large intestine using ultrasound, the doctor may notice not only changes within the intestinal wall, but also outside the object [93]. Thickening of the intestinal wall above 2.5 mm is considered abnormal and classified as a group of lesions resulting from Crohn’s disease. Another aspect that is observed in ultrasound is variable echogenicity and an increased Color Doppler signal, which may indicate excessive tissue blood supply or the inhibition of intestinal peristalsis [93]. In Crohn’s disease, the technique of contrast-enhanced ultrasound (CEUS) is also used. This is a new technique that provides accurate imaging of the perfusion of the intestinal wall and tissues around the holes after intravenous administration of a contrast agent. An oral contrast is most often prepared on the basis of water and polyethylene glycol [94]. Ultrasound examination makes it possible to assess disease activity and complications, characterize stenoses, or verify the effectiveness of pharmacological treatment. Ultrasound is also a tool used to assess blood vessels in the patient’s digestive system and to detect the presence of fluid in the peritoneal cavity [95]. A change in the lymph nodes in the ventral mesentery in many cases is also visible in examination.

According to Dillman et al. [96], it is also possible to perform ex vivo ultrasound examination of collected tissue fragments. It involves assessing the appearance of the intestinal wall and determining its thickness.

Ultrasound examination is one of the safest diagnostic methods of the digestive system in the suspicion and control of Crohn’s disease [94]. It can be performed repeatedly at short intervals without endangering the patient’s health and life. One, and perhaps the only, drawback of this technique is the lack of a standard protocol that should clearly characterize the parameters that are measured and analyzed during an ultrasound examination.

#### 2.6.7. Magnetic Resonance Enterography

Magnetic resonance enterography (MRE) is another diagnostic tool in the assessment of inflammation in Crohn’s disease. Like other imaging techniques, it has its advantages and disadvantages. Its main property is that it does not expose patients to ionizing radiation [97]. This is very important considering the fact that patients with Crohn’s disease require constant control and frequent monitoring of emerging changes. Moreover, this method makes it possible to differentiate inflamed tissues from healthy tissues [98]. Additional advantages include the ideal contrast of soft tissues, mainly epithelial tissue, which is part of the intestinal villi of the small intestine mucosa. Thanks to modern software, it is possible to perform quick scans during the inspection, thus shortening the total examination time. Moreover, over the years, the impact of motion artifacts resulting from, for example, peristaltic movements of the intestines has been minimized, which has significantly improved the quality of the images obtained. In clinical practice, magnetic resonance enterography requires dilating the lumen of the imaged vessels and organs. Therefore, this study requires the administration of a contrast agent, which allows for better visualization of areas within the intestinal walls. It is estimated that the patient should take approximately 1800–2000 mL orally. MR enterography complements endoscopic examination in the analysis of ulcerations, structures and edema [99].

The test protocol depends on the specific clinical entity and is individually prepared for each patient. It depends on the extent of the inflammation and the type of changes occurring. The most commonly used sequences are:

Single Shot Fast Spin Echo (SSFSE);

Balanced gradient-echo sequence in steady state (FIESTA);

Fast Spin Echo (FSE) sequence;

Diffusion-dependent echo-planar imaging (EPI).

These sequences make it possible to locate inflammatory changes, abnormalities in the intestinal walls, pathological edema and anomalies in the physiology of the mucosa and mesenteric vessels.

Images obtained as a result of magnetic resonance imaging are analyzed by radiologists based on an appropriately developed point scale [100]. The following are subject to assessment:

- thickness of the intestinal walls at the sinus [mm];
- degree of contrast enhancement (0–4 points);
- assessment of mural edema in T2-weighted fat-saturated sequences (0–4 points);
- assessment of the peri-mural T2 signal (0–4 points).

Another type of classification is the division of pathological lesions of Crohn’s disease into inflammatory groups, such as: active inflammation, penetrating, fibrostenotic and regenerative-reparative.

Magnetic resonance enterography, despite many advantages and its role in imaging structural changes in abdominal organs, has some limitations. One of them is the inability to visualize early changes in the intestinal mucosa. Additionally, uneven dilation of the intestine with the contrast agent may give the impression of a thickened organ wall.

The range of diagnostic tools for Crohn’s disease is extensive. All techniques presented have their advantages and disadvantages. The choice of a given method depends mainly on the attending physician and the patient’s health condition. The imaging methods used in inflammatory bowel disease diagnosis have been analyzed for several decades. However, it turns out that medical diagnostics is not the only field that can provide information about changes occurring in the patient’s body. Medical biology, which analyzes metalloproteinases, may also be helpful in diagnosing changes that occur as a result of Crohn’s disease.

All developing inflammatory diseases (including Crohn’s disease) cause short-term or permanent cellular changes. Each inflammatory change has a destructive effect on metabolic processes and the action of receptors and enzymes. An example of a Crohn’s disease marker is metalloproteinases, i.e., hydrolytic enzymes, the activity of which increases with the advancement and extent of the disease. Biochemical testing aimed at determining the level of activity of these enzymes is less frequently practiced but is as valuable in diagnostic terms as standard imaging techniques or laboratory tests.

## 3. An Analysis of Matrix Metalloproteinases in Crohn’s Disease—The Role of Biomarkers

### 3.1. Characteristics of Matrix Metalloproteinases

Matrix metalloproteinases (MMPs) are the main group of endopeptidases. Endopeptidases are hydrolytic enzymes from a group of proteases which are responsible, among others, for the degradation of collagen and other proteins [101]. The natural process of collagen destruction is important in the mechanism of tissue self-regeneration [102]. Any pathologies associated with the incorrect functioning of this process are the cause of various diseases. Due to their structure and function, six groups of metalloproteinases are distinguished. These are: collagenases (np. MMP-1, MMP-8, MMP-13 and MMP-18) [103]; gelatinases (np. MMP-2 and MMP-9) [104,105]; Stromelysins (np. MMP-3, MMP-10 and MMP-11) [106,107]; Matrilysins (np. MMP-7 and MMP-26) [108]; membrane-type metalloproteinases (MMP-14, MMP-15, MMP-16, MMP-17, MMP-24 and MMP-25); and a group of other unclassified metalloproteinases (MMP-12, MMP-19, MMP-21 and MMP-28) [109,110,111,112]. The functions of MMP’s are presented in Table 3.

Currently, 23 types of metalloproteinases are known in humans. A common classification distinguishes a number of metalloproteinases, starting with MMP-1 and ending with MMP-28. The general classification does not include: MMP-4, MMP-5, MMP-6 or MMP-22. Metalloproteinases are involved in trophoblast implantation and embryogenesis [113]. They enable bone growth, angiogenesis (development of capillaries), wound healing and tissue regeneration. Gene expression is observed in connective tissue cells, primarily in fibroblasts, but also in neutrophils, monocytes, macrophages and endothelial cells. The biological activity of metalloproteinases is regulated at the level of gene transcription [113]. MMPs are a group of endopeptidases elements. The group is divided into several groups, such as gelatinases, collagenases, stromelysins, matrilysins and membrane-type MMPs. The most frequently characterized are endopeptidases 3, 7, 9 and 11 [113]. The aim of this review is to characterize the four metalloproteinases (3, 7, 9, 11) most frequently characterized and marked in various diseases and cancers.

### 3.2. Review of Studies Characterizing Metalloproteinases in Crohn’s Disease

Under natural (physiological) conditions, MMPs are produced at very low levels and are responsible for normal tissue physiology. The process of production and activation of MMPs is strictly regulated to prevent excessive tissue degradation [114]. Warnaar et al. [115] analyzed the expression of mRNA and the level of metalloproteinases (MMP-1, MMP-3) in tissue sections of the terminal ileum from patients with Crohn’s disease and in a control group. In their results, they reported the following observations: MMP-1 and MMP-3 expression was increased in both pre-stenotic and stenotic tissue. MMP-1 content was elevated in the submucosal and muscle tissue of the prestenotic parts and in the muscle tissue of Crohn’s stenosis samples. MMP-3 was significantly elevated in all tissue samples. Even in the submucosal layer of the proximal tissue of the resection margin, MMP-3 expression was significantly higher than in the control group [115].

In a study conducted by Efsen et al., the proteolytic activity of MMPs in tissues was investigated and the effect of inhibitors (mainly drugs) as MMP inhibitors was investigated [116]. Patients with Crohn’s disease were qualified for the study. High-pressure liquid chromatography was used to measure MMP activity. The results unequivocally indicated that the mean total activity of MMPs was significantly higher in tissues diagnosed with Crohn’s disease compared to fistulas of other disease entities and without signs of other ailments. Both MMP-3 and MMP-9 were elevated compared to non-Crohn’s fistulas.

In turn, Matusiewicz et al. conducted research to estimate MMP-9 concentrations in the sera of patients with Crohn’s disease [117]. A total of 176 patients were qualified for the study group, who were divided into three groups (patients diagnosed with Crohn’s disease, patients with ulcerative colitis, and a control group without signs of disease.) The following observations were presented in the results: concentrations of MMP-9 in were significantly higher in active disease forms. For both CD and UC, serum MMP-9 positively correlated with disease activity. The authors concluded that the assessment of serum MMP-9 concentrations may help to differentiate between active disease form MMP-9 which correlated better with inflammatory and angiogenic parameters in CD rather than in UC [117].

Another determinant in the characteristics of Crohn’s disease is MMP-19. Cervinková et al. [118] analyzed the expression of MMP-19 in CD. The authors identified significant expression of MMP-19 in intact areas of the intestinal epithelium and macrophages. MMP-19 was also abundantly expressed in the endothelium of blood and lymphatic vessels of inflamed intestinal tissue. Significantly high MMP-19 immunoreactivity was also associated with macrophages in inflamed areas and myenteric plexuses [118].

Barberio et al. [119] attempted to test whether serum MMP-3 levels could be considered an early marker of treatment response in CD patients. The study group consisted of 73 patients with CD who were treated with infliximab. The results of the experiment confirmed that MMP-3 levels were usually higher in patients who did not receive the drug than in patients who received drug therapy. The conclusion of the study was that serum MMP-3 determination may be one of the early markers of infliximab treatment [119].

Shamseya et al. [120] investigated the relationship between serum MMP-9 levels and disease activity in patients diagnosed with CD. The research group consisted of 60 patients, 30 of whom were being treated for Crohn’s disease. ELISA was used for the study to determine serum MMP-9 levels. The Mayo scale was used to assess disease activity. The experiment confirmed that the concentration of MMP-9 in the serum was higher in patients with active Crohn’s disease compared to patients with its inactive form. The lowest concentration was found in patients from the control group (i.e., without signs of disease). From the study, it can be concluded that serum MMP-9 can be used to differentiate between active and inactive disease forms [120].

In turn, Gao et al. [121] assessed the expression of MMP-2 and MMP-9 in the intestinal tissue of IBD patients. Tissue samples were collected from 47 patients and divided into three groups (group I—patients with CD, group II—patients with UC, group III—patients without signs of disease, the so-called control group). An enzyme immunoassay (ELISA) was used to assess the activity of MMPs. The experiment showed that MMP-2 and MMP-9 were significantly elevated in sick tissues, with significantly higher levels in severely inflamed tissues. The higher and worse the inflammation, the higher the expression of MMP-9. Immunohistochemistry also showed that MMP-2 was present in the submucosa and MMP-9 in polymorphonuclear leukocytes [121].

According to the literature, up-regulated expression of MMPs plays different functions in pathogenesis, cycles of acute inflammation and resolution and chronic processes such as fibrosis and fistula forms of IBD, including CD. The function of both beneficial and non-beneficial MMPs has not yet been well studied. However, this knowledge has begun to be established for about 6 years [122]. But, in terms of their regenerative role, MMPs have not yet been sufficiently studied in inflammatory bowel disease [123]. MMPs are activated after interactions in the cell–cell and cell–ECM areas or in the mechanism of response to pro-inflammatory cytokines widely expressed in IBD [124,125]. MMPs are involved in the modulation of IBD pathogenesis and cytokines, which are involved in inflammatory processes, and, when developing in the intestine, have the property of increasing MMP levels. As an example, TNFα and bradykinin are able to induce MMP3 expression through a signaling cascade that includes PKC, PKD1 and MEK [126]. Other examples are interleukin 17A (IL17A) and IL17F, which can increase the expression of both MMP-1 and -3 through the myofibroblast. Both IL17 cytokines increased the expression of MMP-1 and -3 [127]. In contrast, other studies suggest a protective function of MMPs in IBD. This is because MMP-2-/- mice have an acute pro-inflammatory response and also a greater susceptibility to disease progression [128]. MMPs are important in many human diseases, but no synthetic MMP inhibitor with a broad spectrum of action has properly completed clinical trials for both the pro-cancer and anti-tumor effects of MMPs in cancer [129]. MMPs (MMP-2, MMP-9, MMP-14) are able to damage the capillary layer and at the same time promote exosmosis of cancer cells. MMP-9 can reduce the level of the IL receptor which is located on the surface of T lymphocytes, and, in addition, it can suppress immunity and promote development of cancer [130,131,132]. In the case of MMP-8, it can directly inhibit tumor metastasis. Side effects of MMP inhibitors, having a broad spectrum of action, usually interfere with MMP-8 tumor inhibition [133].

## 4. Prognosis and Treatment of Crohn’s Disease

In Crohn’s disease, the physiology and functioning of the epithelial barrier are inhibited due to the correlation of various factors, such as the environment, genetic susceptibility or intestinal microbiota [134]. The list of environmental factors that contribute to the increase in the incidence of Crohn’s disease is constantly being supplemented and modified. According to the latest research, smoking cigarettes doubles the risk of disease. Additionally, long-term use of antibiotics during childhood, aspirin, nonsteroidal anti-inflammatory drugs and oral contraceptives may also contribute to the increased incidence. It should be noted that not all environmental factors were analyzed for all countries. Therefore, some of them may have a stimulating effect and lead to faster development of the disease, while in another group of respondents the consequences of these factors will not be visible and the disease will not develop.

According to Freeman et al., the long-term prognosis of Crohn’s disease is almost stable [135]. In turn, based on observational studies conducted by Martínez Sánchez et al., perianal diseases are a common consequence of Crohn’s disease. This results in a worse prognosis and more often requires biological treatment [136]. Approximately 50% of patients require surgical treatment within 10 years of diagnosis, and 70–80% during their lifetime. Patients suffering from Crohn’s disease have a significantly increased risk of developing colon and small intestine cancer. Pharmacological treatment of Crohn’s disease is divided into specific and symptomatic treatment. Specific pharmacological treatment includes preparations such as: glucocorticosteroids—administered orally; prednisone or prednisolonel budesonide; and, in cases of highly active disease, intravenous hydrocortisone. Other types of drugs are immunosuppressive drugs used in the treatment of maintaining remission, i.e., azathioprine and methotrexate. In turn, biological drugs include infliximab, adalimumab, vedolizumab and ustekinumab. During treatment, the doctor may also recommend antibiotics, e.g., for the treatment of perianal fistulas. In turn, in symptomatic treatment, painkillers are used, such as metamizole or, e.g., tramadol. Treatment of Crohn’s disease is chronic; it involves preventing relapses and alleviating the course of exacerbations. A complete cure is probably impossible, but there are mild forms of the disease without exacerbations. A rare long-term consequence of inflammatory bowel diseases is colorectal cancer (approximately 1.5% of patients). Factors increasing the risk of its occurrence include: long duration of the disease and involvement of a significant part of the large intestine.

## 5. Conclusions

One of the inflammatory bowel diseases is Crohn’s disease. Crohn’s disease is a disease that cannot be fully cured. In many cases, it is chronic, i.e., recurrent. All therapeutic measures undertaken by doctors are aimed at stopping the development of the disease and minimizing the occurrence of any potential “side effects” resulting from the developing disease. The range of diagnostic tools for Crohn’s disease is extensive. All techniques presented have their advantages and disadvantages. The choice of a given method depends mainly on the attending physician and the patient’s health condition. The imaging methods used in inflammatory bowel diseases have been analyzed for several decades. The chronicity of Crohn’s disease causes severe life discomfort for patients, therefore the use of a non-invasive diagnostic method is very important in the context of the entire diagnostic and therapeutic procedure. Metalloproteinase analysis also proves useful in the diagnosis of Crohn’s disease. The most frequently analyzed metalloproteinases in Crohn’s disease are: MMP-1, MMP-3, MMP-7, MMP-8 and MMP-9. Their presence and increased value inform about emerging and ongoing inflammation.

## Figures and Tables

**Table 1:** Verification of intestinal changes in Crohn’s disease, based on the CGHAS disease activity index.

Change	Evaluation	Point Verification
**Degree of epithelial damage**	Lack	0
	Focal change	1
	Extensive changes	2
**Structural changes**	Natural	0
	Moderate	1
	To a severe degree	2
**Number of neutrophils in the lamina propria**	The number is correct	0
	Moderate growth	1
	Significant increase	2
**Presence of neutrophils in the epithelium**	Epithelial surface	1
	Inflammation	2
	Abscesses	3
**Ulcers**	Occur	0
	Do not occur	1
**Number of mononuclear cells in the lamina propria**	The number is correct	0
	Moderate growth	1
	Significant increase	2
**Presence of ringworm**	Occurs	0
	Absent	1
**Percentage of biopsy fragments with inflammatory changes in the total material**	<33%	1
	33%−66%	2
	>66%	3

**Table 2:** Typical changes analyzed in a study using computed tomography.

Type of Change	Characteristics of the Analysis
Intra-abdominal abscesses	Presence of lesion, physiological assessment
Strictures, fistulas	Determining the number
Thickening of the intestinal wall	Assessment of the degree and extent of thickening
Increased density of mesenteric fat	Physiological assessment
Intestinal bleeding	Assessment of the extent of bleeding
Strengthening the mucous membrane in the ileocecal area	Assessment of the change and its extent
Ulcers	Extensiveness analysis
Enlarged lymph nodes	Attendance assessment

**Table 3:** MMP’s functions.

MMPs Name	Function
MMP-1	inhibition of fibrosis
MMP-2	inhibition of angiogenesis, influence on the epithelial barrier function, inhibition of fibrosis
MMP-3	outflow to the production of Endostatin
MMP-7	alpha-defensin activation, chemokine expression, ulcer healing, Endostatin production
MMP-8	neutrophil infiltration
MMP-9	Chemokine expression, neutrophil infiltration, production of anti-angionenic factors, processing, VEGF-A activation, inhibition of goblet cell differentiation, inhibition of fibrosis
MMP-10	healing of ulcers
MMP-13	TNF-alpha activation and Endostatin production
MMP-14	ability to activate proMMP-2
MMP-15	ability to activate proMMP-2
MMP-16	ability to activate proMMP-2
MMP-17	participation in the breakdown of the extracellular matrix, reproduction and tissue remodeling
MMP-19	Activity in the vessels of the synovial membrane
MMP-20	Endostatin production
MMP-23	presence in reproductive tissues
MMP-24	I group of membrane proteins
MMP-25	regulate innate immunity acting over proinflammatory cytokines, chemokines, and other immune-related proteins
MMP-28	wound healing

## Data Availability

Data are contained within article.
